# In search of a reliable electrophysiological marker of oculomotor inhibition of return

**DOI:** 10.1111/psyp.12245

**Published:** 2014-06-27

**Authors:** Jason Satel, Matthew D Hilchey, Zhiguo Wang, Caroline S Reiss, Raymond M Klein

**Affiliations:** a School of Psychology, Faculty of Science, University of Nottingham Malaysia CampusSemenyih, Malaysia; b Department of Psychology & Neuroscience, Faculty of Science, Dalhousie UniversityHalifax, Nova Scotia, Canada; c Center for Cognition and Brain Disorders, Hangzhou Normal UniversityHangzhou, China

**Keywords:** Inhibition of return, Cueing, Event-related potentials, Oculomotor activation, Sensory and motor processing, Eye movements

## Abstract

Inhibition of return (IOR) operationalizes a behavioral phenomenon characterized by slower responding to cued, relative to uncued, targets. Two independent forms of IOR have been theorized: input-based IOR occurs when the oculomotor system is quiescent, while output-based IOR occurs when the oculomotor system is engaged. EEG studies forbidding eye movements have demonstrated that reductions of target-elicited P1 components are correlated with IOR magnitude, but when eye movements occur, P1 effects bear no relationship to behavior. We expand on this work by adapting the cueing paradigm and recording event-related potentials: IOR is caused by oculomotor responses to central arrows or peripheral onsets and measured by key presses to peripheral targets. Behavioral IOR is observed in both conditions, but P1 reductions are absent in the central arrow condition. By contrast, arrow and peripheral cues enhance Nd, especially over contralateral electrode sites.

Efficient sampling of the environment requires a mechanism that biases orienting against previously inspected locations (Itti & Koch, 2001; Klein, 1988). A mechanism hypothesized to fulfill this role was first proposed by Posner and Cohen (1984) following behavioral observations from the classic spatial cueing paradigm (Posner, 1980). In a seminal experiment, Posner and Cohen (1984; see also Posner, Cohen, & Rafal, 1982) instructed participants to maintain fixation and make key press responses to visual targets at, or opposite, the location of a transient, spatially uninformative visual stimulus (a cue). When the delay between the cue and target exceeded 300 ms, response times (RTs) to targets appearing at the same location as the cue were longer than those appearing at distance-matched locations in the opposite visual field.

Subsequent work revealed that (a) the effect (i.e., slowed responding to cued locations) is closely linked to the midbrain saccade systems that drive orienting responses, specifically the superior colliculus (Posner, Rafal, Choate, & Vaughan, 1985, Experiment 1; Sapir, Soroker, Berger, & Henik, 1999; Sereno, Briand, Amador, & Szapiel, 2006), (b) orienting responses are biased against previously cued locations (Posner et al., 1985, Experiment 2; Klein & MacInnes, 1999; Bays & Husain, 2012; MacInnes, Hunt, Hilchey, & Klein, 2014; see Wang & Klein, 2010, for review), whereas perceptual arrival time is not (Posner et al., 1985, Experiment 2; Schmidt, 1996), and (c) most crucially for the present investigation, oculomotor responses are sufficient (i.e., peripheral cueing is not necessary) to generate the effect (Posner et al., 1985, Experiment 3; Chica, Klein, Rafal, & Hopfinger, 2010; Rafal, Calabresi, Brennan, & Scioloto, 1989; Taylor & Klein, 2000). Reflecting Posner et al.'s (1985) proposal that orienting was inhibited from returning to the location of a previous orienting response, this phenomenon and accompanying theoretical framework was labeled *inhibition of return* (IOR; see Hilchey, Klein, & Satel, 2014, for a review).

## Neural Correlates of IOR

A wide range of approaches and tools [spanning behavior (Posner & Cohen, 1984), development (Clohessy, Posner, Rothbart, & Vecera, 1991), lesion patients (Sapir et al., 1999), functional magnetic resonance imaging (fMRI; Mayer, Dorflinger, Rao, & Seidenberg, 2004), extracellular recording (Dorris, Klein, Everling, & Munoz, 2002), transcranial magnetic stimulation (TMS; van Koningsbruggen, Gabay, Sapir, Henik, & Rafal, 2009), and electroencephalography (EEG; McDonald, Ward, & Kiehl, 1999)] have been used to further evaluate the neural mechanisms underlying IOR when responses to visual signals are discouraged (for a review, see Klein, 2004). Among the neuroimaging techniques that have been applied to IOR, EEG methods and the event-related potentials (ERPs) they measure have featured prominently in many investigations involving human subjects (for reviews, see Prime & Ward, 2006; Satel, Hilchey, Wang, Story, & Klein, 2013). Using ERPs to investigate attentional phenomena, a number of studies have revealed that several relatively early ERP components can be modulated by attention. The amplitude of P1, the first positive peak that is usually observed around 100 ms after a visual onset, has been shown to increase when a stimulus is attended and to decrease when a stimulus is inhibited or ignored (e.g., Luck, Hillyard, Mouloua, Woldorff, Clark, & Hawkins, 1994).

Consistent with the notion that IOR biases attentional orienting by affecting/reducing the strength of the cued signal (e.g., Posner & Cohen, 1984; Posner, Cohen, Choate, Hockey, & Maylor, 1984; Posner et al., 1982; but see Posner et al., 1985), several studies have demonstrated that behavioral IOR is often accompanied by a reduction in target-elicited P1 amplitude for cued targets in spatial cueing paradigms in which the observer is instructed to maintain fixation and make a key press response to a peripherally presented visual target (McDonald et al., 1999, Experiment 3; Prime & Ward, 2004; van der Lubbe, Vogel, & Postma, 2005; Wascher & Tipper, 2004, Experiment 1; Prime & Jolicoeur, 2009b; Prime & Ward, 2006; Satel et al., 2013; Tian & Yao, 2008). However, P1 reductions for cued targets are not always observed in conjunction with IOR (Hopfinger & Mangun, 2001; McDonald et al., 1999, Experiment 2; Prime & Ward, 2006; van der Lubbe et al., 2005, Experiment 3), and are sometimes observed without any behavioral evidence for IOR (Chica & Lupianez, 2009; Doallo et al., 2004; Hopfinger & Mangun, 1998; Wascher & Tipper, 2004). Satel et al. (2013) demonstrated that, although such target-elicited P1 reductions were correlated with the magnitude of the IOR effect when the oculomotor system was forbidden from responding to peripheral onset events, there was no such correlation when saccades were made to the cues. As such, and in line with our motivation for conducting the present investigation (discussed in the next section), it remains possible—if not likely—that the neural substrates underlying IOR are different when oculomotor responding is imperative and not actively discouraged.

Another ERP component that has been associated with IOR in previous work is the Nd (negative difference) component, which is a negative deflection in the ERP waveform in the time period between about 220–300 ms poststimulus (Prime & Ward, 2006). Many studies using a cue-target IOR paradigm have observed Nds along with behavioral IOR (McDonald et al., 1999; Prime & Jolicoeur, 2009b; Prime & Ward, 2004, 2006; Satel et al., 2013; Wascher & Tipper, 2004), potentially reflecting an association with IOR (Satel, Wang, Hilchey, & Klein, 2012). Because the Nd component occurs later in time than the P1 component, its modulation by cueing suggests that IOR affects later processing, possibly in addition to providing a sensory gain control function as is often inferred from the observed P1 modulation. Previous work using a go/no-go cueing paradigm (Prime & Ward, 2006) demonstrated that the Nd effect was absent on trials when no response was required, suggesting that the Nd effect may be associated with response selection and/or response execution.

## Two Forms of IOR

Although researchers often assume that IOR and its neural substrates generalize from tasks in which eye movements are forbidden to real-world search, variations on the classic spatial cueing paradigm have convincingly demonstrated that the mechanisms responsible for—and the effects of—IOR are fundamentally different depending on whether the task permits or requires oculomotor responses (e.g., Bourgeois, Chica, Migliaccio, de Schotten, & Bartolomeo, 2012; Hilchey et al., 2014; Hunt & Kingstone, 2003; Kingstone & Pratt, 1999; Satel et al., 2013; Smith, Schenk, & Rorden, 2012; Sumner, Nachev, Vora, Husain, & Kennard, 2004; Taylor & Klein, 2000; see Klein & Hilchey, 2011, for review). The effect of IOR in tasks forbidding oculomotor responding is nearest the perceptual—or input—end of the processing continuum, whereas the effect of IOR in tasks requiring or permitting oculomotor responding is nearer the motoric—or output—end of the processing continuum (see also Taylor & Klein, 2000; Hilchey et al., 2014). Despite this, an overwhelming majority of research reports (i.e., those reviewed above) explore the neural substrates or markers of IOR administered in tasks in which eye movements are discouraged, an approach which may partially account for why most extant electrophysiological data tend toward an association between IOR and input-based processes. Far fewer research reports, however, have sought to identify neural signatures of IOR in tasks for which eye movements are required or permitted, and the effect of IOR is thus output based.

Because IOR is thought to generalize to natural search (see Wang & Klein, 2010, for review), and because natural search represents a state in which the oculomotor system is free, if not required, to respond to relevant inputs (e.g., Smith & Henderson, 2011), we are concerned that the electrophysiological signature of IOR, obtained from the classic covert orienting paradigm, is unlikely to generalize much beyond the laboratory. The research reported here was designed to remedy this weakness. Our objectives are threefold:

1. We explore ERPs in a cueing paradigm that generates an output-based form of IOR, more likely to be expressed during natural search, in an effort to unveil a reliable electrophysiological marker of the phenomenon. In addition to causing IOR with a saccadic response to a peripherally presented visual stimulus (as in Satel et al., 2013), we also cause IOR with an eye movement to a left or right pointing arrow at fixation (e.g., Chica et al., 2010; Posner et al., 1985; Rafal et al., 1989; Taylor & Klein, 2000) so as to measure IOR in a condition without repeated stimulation of the sensory pathways. The central arrow condition here is the first of its kind presented in the EEG literature on IOR. This departure from common practice in EEG research on IOR is noteworthy because we will be able to visualize the electrophysiological signature of IOR under conditions in which cues and targets are not spatially coincident. Importantly, because arrows that are not associated with a response seldom, if ever, generate IOR or IOR-like phenomena at late cue-target onset asynchronies (see Hilchey, Satel, Ivanoff, & Klein, 2013, for a review and experiments demonstrating how arrows generate IOR or IOR-like effects), we are assured that the IOR effect in this case is fundamentally caused by the saccadic eye movement. This condition is thus likely to yield the form of IOR that Posner et al. (1985) generated (Experiment 3) and conceptualized in their flagship paper. That is, IOR is a long-lasting (> 1 s) response bias, occurring in the aftermath of oculomotor response activation, that operates in the service of novelty seeking.
2. We remain dedicated to further exploring the contentious relationship between IOR and target-elicited P1 activation. There is little reason to suspect a relationship between output-based IOR and target-elicited P1 activation given that the effect is decidedly postperceptual in nature (e.g., Taylor & Klein, 2000). Despite this, and although there was no correlation between the target-elicited P1 reduction and the magnitude of IOR in our previous investigation on eye movements (Satel et al., 2013), the target-elicited P1 was nevertheless reduced when the target occurred at the same location as the cue. Such an effect may merely be the result of repeatedly stimulating sensory pathways and may vanish when IOR is caused by an eye movement to an arrow. If this predicted pattern of results were borne out, the implication would provide further evidence (see also Satel et al., 2012) that modulation of the target-elicited P1 is neither necessary, nor sufficient, for the output-based form of IOR.
3. Finally, we will explore modulations of the target-elicited Nd ERP component by IOR in an effort to determine whether this later component is a more reliable neural correlate of output-based IOR.

## Method

### Participants

Twenty-one university students took part in this experiment in exchange for course credit or monetary incentive. They were recruited from the participant pool in the Department of Psychology at Dalhousie University. They all reported normal or corrected-to-normal vision. Data from one subject were excluded from analysis due to excessive anticipatory responses. The average age of the remaining participants (15 female, 5 male) was 20.4 years.

### Stimuli and Apparatus

All stimuli were drawn in white on a black background and presented on a 19” Asus LCD monitor controlled by an AMD Athlon 64-bit personal computer. Manual response times (MRTs)—to targets—were collected with a Microsoft keyboard. Participants sat in a darkened and electromagnetically shielded room, with their heads stabilized by a chin rest located 57 cm away from the computer screen. Eye position was monitored throughout the experiment using a video-based EyeLink 1000 eye tracking system sampling at 250 Hz. Saccadic response times (SRTs)—to cues—were recorded with the eye tracker. EEG data were recorded continuously at 256 Hz with a BioSemi ActiveTwo amplifier system, which used 64 Ag-AgCl electrodes mounted in an elastic cap according to the International 10–20 system. Six electrodes were also placed at the outer canthi of the eyes as well as above and below the left eye, and on the mastoids. Two additional electrodes (CMS: common mode sense and DRL: driven right leg; positioned on either side of electrode site Oz in the BioSemi 64-channel cap) served as recording reference and ground.

### Design and Procedure

**Figure 1:**
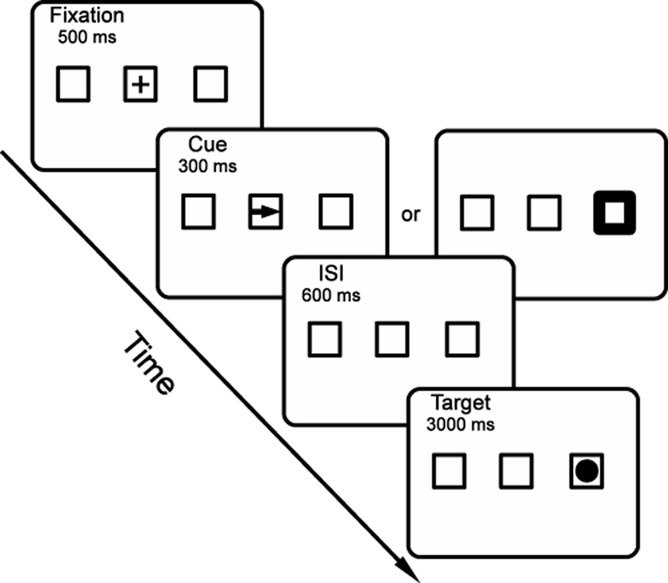
Sequence of events in a sample trial. Although uninformative about the location of the upcoming peripheral target, the cue (whether central or peripheral) called for a saccade to the indicated peripheral box, and participants were instructed to saccade back to the original fixation as quickly as possible. The target appears at the cued (and recently fixated) location.

The present experiment adopted a 2 (Condition: peripheral cue vs. central cue) × 2 (Cueing: cued vs. uncued) within-subject design (see Figure 1). The cue was either an arrow at fixation or a peripheral onset, and the target (always a peripheral onset) either appeared at a cued or uncued location. All four trial types were mixed and presented to participants in a random order. Participants made saccadic eye movements to the cues and manual localization responses to the targets. The experimental procedure was an extension of the paradigm used by Taylor and Klein (2000). Each trial began with a self-paced oculomotor drift correction to a red cross at the center of the display. Then, three landmark boxes (4.5° × 4.5°, visual angle) were added to the display, one at the center and the other two on either side, 8.7° away from center. Participants maintained fixation on a cross in the central box for 500 ms. Then, for 300 ms, a nonpredictive cue appeared as either a thickening of one of the peripheral boxes or as an arrow in the central box pointing toward one of the peripheral boxes. Participants were instructed to move their eyes as quickly and accurately as possible to the box indicated by the cue, and then to move their eyes back to the central box within 600 ms. In contrast to Taylor and Klein (2000), a cue-back stimulus in the central box was not used in this experiment in order to eliminate the potential confound of multiple stimulations of the same retinotopic location occupied by uncued targets (see Wang, Satel, & Klein, 2012, for a discussion of this issue) and to enhance the ecological validity of our design. After a cue-target onset asynchrony of 900 ms, a target (a bright disk with a diameter of 2.4°) appeared in one of the two peripheral boxes. Participants were instructed to make a manual localization response, using the z and diagonal (/) keys for targets appearing in the left and right boxes, respectively. All participants were tested with four blocks of 100 trials. Practice trials were provided at the beginning of each experimental session until the participant felt comfortable performing the task.

To obtain as many useful trials as possible, an online algorithm was implemented to recycle incorrectly completed trials. Saccade amplitude was measured as the distance between the initial gaze coordinates and the saccadic end point by the eye tracker online. If a saccade of more than 3° was detected during the fixation period, the trial was aborted and an error message with instructions to maintain fixation was presented. In addition, the participant had 600 ms in total to move their eyes to within 3° of the center of the peripheral location indicated by the cue and then return to fixation. Failure to do so resulted in the trial being aborted with an error message indicating that the required eye movements were not completed correctly. All aborted trials were retested throughout the rest of the experiment in random order.

### Analysis of EEG Data

The EEG data were analyzed with the EEGLAB toolbox (Delorme & Makeig, 2004) in MATLAB. The EEG data were digitally filtered with a high-pass filter of 0.1 Hz and a low-pass filter of 30 Hz. Bad electrodes were identified as any electrode that had greater than ± 100 microvolt deflections on more than 60% of trials and were not used in the artifact rejection procedure. Data were then rereferenced to the average of all electrodes and segmented into epochs starting 250 ms before and ending 400 ms after target appearance. After performing a 100-ms baseline correction, trials with excessive artifacts (± 75 microvolts) were excluded from further analyses. Trials with incorrect behavioral responses or any incorrect eye movements were also excluded from further analysis.

The parieto-occipital P1, N1, and Nd ERP components were considered in our statistical analyses. These components were quantified by measuring each participant's mean EEG amplitude at ipsilateral and contralateral parieto-occipital electrodes (PO7/8) over a 20-ms (P1/N1) or 40-ms (Nd) time window centered around the peak of the component. The peaks were identified in each individual's average waveform (averaged over all trial types) as the maximal point of the P1 and N1 components or in the cued versus uncued difference wave for the Nd component. PO7/8 electrodes were selected for analysis based on the results of previous work investigating similar paradigms with similar stimuli for consistency of analyses and the ability to make general comparisons across studies (e.g., Satel et al., 2013). Based on the literature and our prior work, we have analyzed the ipsilateral and contralateral electrodes separately for P1 and N1 components, and we perform planned comparisons on all components separately to investigate cueing effects in each condition.

## Results

### Behavioral Performance

Trials with incorrect eye movements during the trials (3.40%), as identified by the eye tracker, were removed prior to analyses. Trials with excessive ERP artifacts during the extracted epochs were also removed before all statistical analyses (10.98%). Trials with no response (1.59%), incorrect responses (wrong key, 1.39%), anticipatory responses (MRTs faster than 200 ms; 1.51%), or slow responses (MRTs slower than 650 ms; 0.50%) were also excluded from analyses. The mean correct MRTs for each condition are presented in Table 1.

**Table 1:** Mean MRTs and ERP Component (P1, N1, and Nd) Amplitudes for Cued and Uncued Trials

	Central cue			Peripheral cue		
Cued		Uncued	Cueing effect	Cued	Uncued	Cueing effect
MRT (ms)	332.54 (42.70)	313.73 (40.22)	18.8***	352.17 (47.41)	299.82 (36.74)	52.4***
P1 (μV)						
Ipsi	0.85 (1.83)	0.96 (1.95)	−0.11	1.04 (1.56)	1.93 (2.23)	−0.89*
Contra	1.32 (2.07)	1.08 (2.16)	0.24	1.63 (2.40)	1.42 (2.39)	0.21
N1 (μV)						
Ipsi	−3.91 (2.37)	−3.96 (2.16)	0.05	−2.86 (2.15)	−3.57 (2.48)	0.71^*^
Contra	−3.47 (2.70)	−3.04 (2.82)	−0.43	−2.65 (2.75)	−3.27 (2.79)	0.62
Nd (μV)						
Ipsi	−3.87 (3.07)	−5.04 (3.24)	1.17**	−2.69 (3.50)	−3.35 (2.97)	0.66
Contra	−1.02 (2.57)	−2.41 (3.07)	1.39*	0.58 (2.63)	−2.05 (2.84)	2.63***

*Note*. Standard deviations are presented in parentheses.

*** *p* < .001.

** *p* < .01.

* *p* < .05.

A repeated measures analysis of variance (ANOVA) on the MRTs, with factors condition (peripheral cue vs. central cue) and cueing (cued vs. uncued), revealed a main effect of cueing, *F*(1,19) = 69.929, *p* < .001, 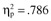, suggesting that IOR was observed. The main effect of condition did not reach significance, *F*(1,19) = 1.759, *p* = .201, 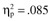, because there was no reliable difference in MRTs to targets preceded by peripheral versus central cues. However, the interaction of Cueing × Condition did reach significance, *F*(1,19) = 44.261, *p* < .001, 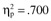. Planned comparisons demonstrated that this interaction was a result of greater IOR when the cue was peripheral (52.4 ms), *t*(19) = 9.236, *p* < .001, than when it was central (18.8 ms), *t*(19) = 4.590, *p* < .001, a pattern that was not observed by Taylor and Klein (2000).

Participants in the present investigation were required to make an eye movement to the cues. As discussed in the introduction, this manipulation was critical to generating the output-based form of IOR (Taylor & Klein, 2000). Analysis of the eye movement data revealed that, as expected, SRTs to central arrow cues were longer than those to peripheral onset cues, *t*(18) = 13.25, *p* < .001, with 243 ms and 200 ms SRTs for central and peripheral cue conditions, respectively. Saccades to central cues were less accurate than those to peripheral cues, *t*(18) = 3.30, *p* < .01, with the landing positions missing the center of the cue-indicated target boxes by 1.73° and 1.38°, respectively (see Hilchey et al., 2014, for a discussion of this issue and similar results).

### Event-Related Potentials (ERPs)

**Figure 2:**
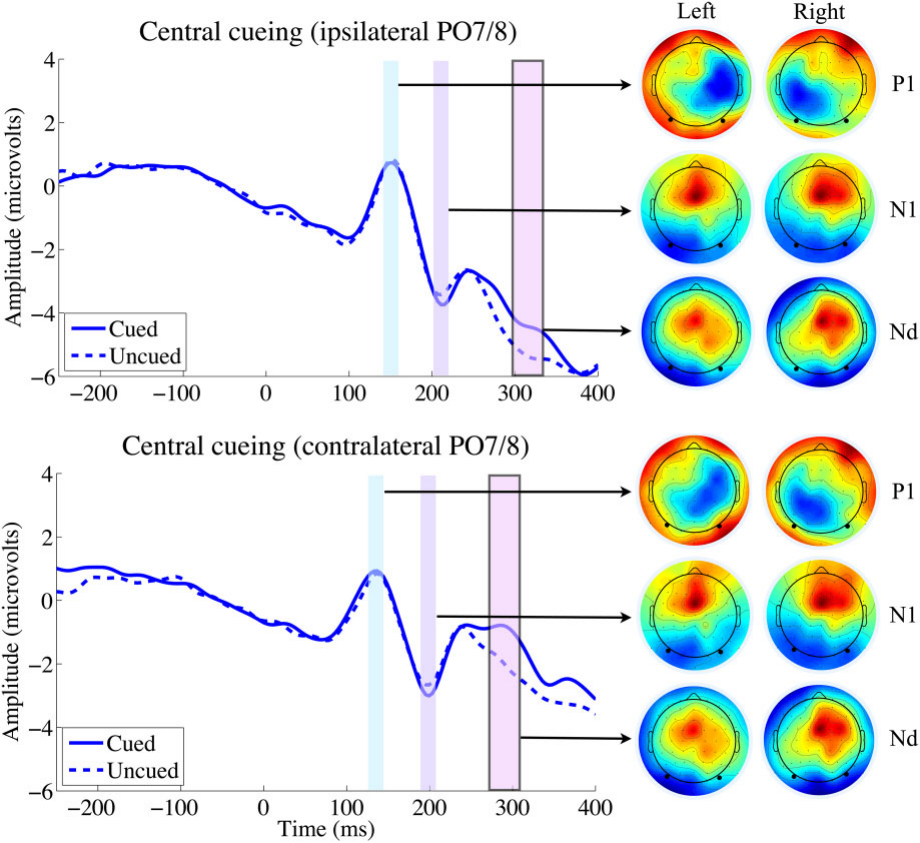
Target-elicited ERP waveforms and topographic maps for the central cue condition recorded from ipsilateral and contralateral parieto-occipital electrodes (PO7/8; indicated as solid black dots in the topographic maps). Time windows in the ERP plots indicate the windows used to calculate ERP component amplitudes (see text for details). Time windows with a solid outline represent significant differences between cued and uncued ERP components (*t* tests, *p* < .05); for central cueing, only the Nd component showed a cueing effect, for both ipsilateral and contralateral electrodes. Topographic heat maps associated with each time window/ERP component for targets appearing on the left and right sides are presented, with red indicating most positive activation and blue indicating most negative activation.

**Figure 3:**
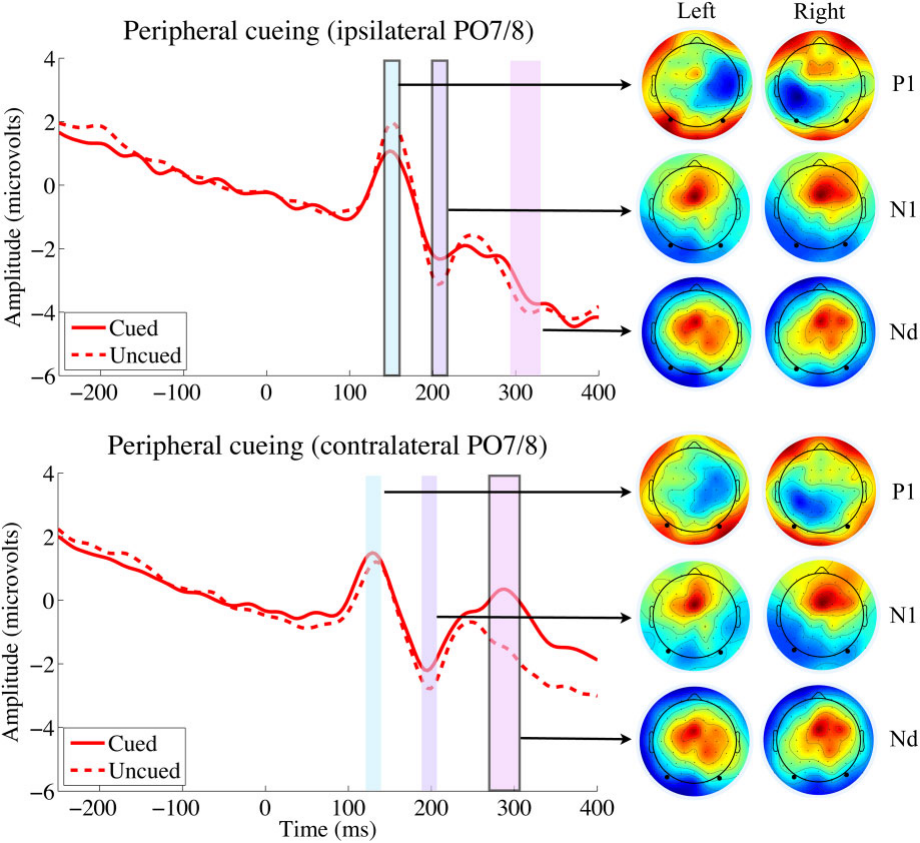
Target-elicited ERP waveforms and topographic maps for the peripheral cue condition recorded from ipsilateral and contralateral parieto-occipital electrodes (PO7/8; indicated as solid black dots in the topographic maps). Time windows in the ERP plots indicate the windows used to calculate ERP component amplitudes (see text for details). Time windows with a solid outline represent significant differences between cued and uncued ERP components (*t* tests, *p* < .05); for peripheral cueing, only the ipsilateral P1 and contralateral Nd components showed a cueing effect. Topographic heat maps associated with each time window/ERP component for targets appearing on the left and right sides are presented, with red indicating most positive activation and blue indicating most negative activation.

Statistical analyses were performed on the P1, N1, and Nd ERP component amplitudes, which were derived from EEG data as described above. The mean ERP amplitudes for each trial type are presented in Table 1, and the grand-averaged ERP waveforms are presented in Figure 2 (central cueing) and Figure 3 (peripheral cueing). Repeated measures ANOVAs and planned comparisons were performed separately for each ERP component and are presented below.

#### P1 component

For ipsilateral electrodes, the ANOVA with factors condition and cueing^1^ revealed a main effect of condition, *F*(1,19) = 6.875, *p* = .017, 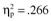, due to overall larger target-elicited P1s in the peripheral cueing condition. There was a marginally significant main effect of cueing, (F(1,19) = 3.844, *p* = .065, 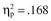, due to overall larger P1s when targets were uncued. The interaction of Cueing × Condition was also marginally significant, *F*(1,19) = 3.554, *p* = .075, 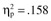. This marginal interaction reflects larger cueing effects on the ipsilateral P1 component for peripherally cued targets than for centrally cued targets. Planned comparisons on the ipsilateral electrodes showed that there were indeed cue-induced reductions to target-elicited P1 components in the peripheral cue condition (−0.89 μV), *t*(19) = 2.496, *p* = .011, whereas no such difference was observed in the central cue condition (−0.11 μV), *t*(19) = 0.374, *p* = 0.356. For contralateral electrodes, the ANOVA with factors condition and cueing revealed no main effect of condition, *F*(1,19) = 1.682, *p* = .210, 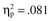, or cueing, *F*(1,19) = 1.303, *p* = .268, 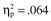, and no interaction of Cueing × Condition, *F*(1,19) = 0.004, *p* = .949, 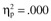. Planned comparisons on the contralateral electrodes did not show any cue-induced effects on target-elicited P1 components in the peripheral cue condition (0.24 μV), *t*(19) = 0.646, *p* = .526, or the central cue condition (0.21 μV), *t*(19) = 1.137, *p* = .270.

#### N1 component

For ipsilateral electrodes, the ANOVA with factors condition and cueing^2^ revealed a main effect of condition, *F*(1,19) = 8.323, *p* = .009, 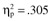, but there was no main effect of cueing, *F*(1,19) = 1.833, *p* = .192, 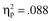. The interaction of Cueing × Condition was not significant, *F*(1,19) = 2.533, *p* = .128, 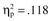. Planned comparisons on the ipsilateral electrodes revealed cue-induced enhancements to target-elicited N1 components in the peripheral cue condition (0.71 μV), *t*(19) = 1.888, *p* = .037, whereas no such difference was observed in the central cue condition (0.05 μV), *t*(19) = 0.878, *p* = .439. For contralateral electrodes, the ANOVA with factors condition and cueing revealed no main effect of condition, *F*(1,19) = 1.816, *p* = .194, 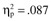, or cueing, *F*(1,19) = 0.061, *p* = .807, 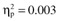, but there was a significant interaction of Cueing × Condition, *F*(1,19) = 4.471, *p* = .048, 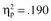. Planned comparisons on the contralateral electrodes showed marginally significant cue-induced enhancements to target-elicited N1 components in the peripheral cue condition (0.62 μV), *t*(19) = 1.487, *p* = .0765, whereas no such difference was observed in the central cue condition (−0.43 μV), *t*(19) = 0.888, *p* = .193.

#### Nd component

An exploratory omnibus ANOVA was performed on the Nds with factors condition, cueing, and laterality, which revealed a main effect of cueing, *F*(1,19) = 24.618, *p* < .001, 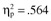, due to larger cued than uncued Nds. There was a main effect of condition, *F*(1,19) = 37.118, *p* < .001, 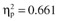, due to overall more negative Nds in the central condition. The main effect of laterality was also significant, *F*(1,19) = 21.091, *p* < .001, 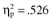, due to overall more negative Nds ipsilaterally. There was an interaction of Cueing × Laterality, *F*(1,19) = 11.060, *p* = .004, 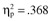, due to larger cueing effects at the contralateral electrodes. The interaction of Cueing × Condition, *F*(1,19) = 0.573, *p* = .458, 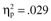, was not significant, and the interaction of Condition × Laterality was marginally significant, *F*(1,19) = 3.185, *p* = .090, 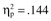. Importantly, all effects were qualified by a three-way interaction of Cueing × Condition × Laterality, *F*(1,19) = 10.083, *p* = .005, 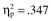. To decompose this interaction, the ipsilateral and contralateral electrodes were subjected to separate ANOVAs.

For ipsilateral electrodes alone, the ANOVA with factors condition and cueing revealed a main effect of condition, *F*(1,19) = 34.061, *p* < .001, 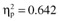, and a main effect of cueing, *F*(1,19) = 8.038, *p* = .011, 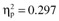. The interaction Cueing × Condition was not significant, *F*(1,19) = 1.013, *p* = .327, 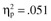, demonstrating that there were equivalent cueing effects on Nds in both the peripheral and central cueing conditions. Planned comparisons on the ipsilateral electrodes showed that there were cue-induced enhancements to target-elicited Nd components in the central cue condition (1.17 μV), *t*(19) = 2.971, *p* = .004, but they were not quite significant in the peripheral cue condition (0.66 μV), *t*(19) = 1.55, *p* = .069. For contralateral electrodes alone, the ANOVA with factors condition and cueing revealed a main effect of cueing, *F*(1,19) = 19.265, *p* < .001, 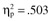, and a main effect of condition, *F*(1,19) = 32.444, *p* < .001, 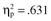. The interaction between Cueing × Condition was marginally significant, *F*(1,19) = 1.956, *p* = .178, 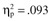, since cueing effects were stronger with peripheral cues. Planned comparisons on the contralateral electrodes showed that there were cue-induced enhancements to target-elicited Nd components in both the peripheral cue condition (2.63 μV), *t*(19) = 6.325, *p* < .001, and in the central cue condition (1.39 μV), *t*(19) = 2.731, *p* = .0065.

## Discussion

Previous EEG studies of IOR have demonstrated that IOR is often accompanied by an amplitude reduction of early ERP components (P1 and N1 modulations) when fixation is maintained and peripheral stimuli (both cues and targets) are used to evoke and measure IOR. According to the two-forms theory of IOR proposed by Taylor and Klein (2000), the IOR effects revealed in these ERP studies were input based by nature only to the extent that observers were actively suppressing the natural tendency to execute saccadic eye movements toward peripherally occurring signals. In point of fact, interpretation of previous EEG data in the covert orienting paradigm is largely consistent with Taylor and Klein's (2000) input-based form of IOR (e.g., Prime & Ward, 2006). In Satel et al. (2013), the magnitude of the more input-based form of IOR was significantly correlated with cue-modulated reductions in early sensory ERP components, whereas the output-based form was not. Reinforcing this point—in the present investigation, wherein the output-based form was generated by a saccade to peripheral and central cues, we obtained IOR whether (peripheral cue) or not (central cue) a target-elicited P1 reduction was observed.

The primary goal of the present study was to adapt the oft-used covert spatial orienting paradigm to elicit a form of IOR that is more likely to be operating during real-world search tasks (i.e., output-based IOR when the effect is not generated by transient flashes of light in peripheral vision) in an effort to identify a robust electrophysiological or neural substrate for oculomotor IOR. The key behavioral and electrophysiological results of the present investigation can be summarized and interpreted as follows: (1) Although behavioral IOR was observed in both peripheral and central cue conditions, the effect was larger when generated by peripheral than central cues, a finding that hints, in conjunction with finding #2 (see below), at the possibility that peripherally generated and peripherally measured IOR involves a sensory component (Satel, Wang, Trappenberg, & Klein, 2011; Wang et al., 2012; but see Hilchey, Klein, & Ivanoff, 2012; Hilchey et al., 2014; Taylor & Klein, 2000). (2) Cue-induced reductions in target-elicited P1 activation were observed only in the peripheral (not central) cue condition, suggesting that the effect of IOR when generated by a central arrow and measured by a peripheral signal is unlikely to be on low-level sensory processes (Taylor & Klein, 2000), whereas the effect generated and measured by peripheral signals might be (Wang et al., 2012). (3) The Nd component was modulated by cueing in both peripheral and central cue conditions at contralateral and ipsilateral electrode sites, although the effect of the peripheral cue was considerably weaker at ipsilateral electrode sites than at contralateral sites. These novel findings, when considered with finding #2, suggest that Nd is independent of the low-level perceptual effects detected by target-elicited P1 and N1 activation (but see alternative hypotheses made by, e.g., Eimer, 1994; McDonald et al., 1999, on data collected from a spatial cueing paradigm in which the cue and target always occurred at the same location) and point to Nd as a more robust electrophysiological marker of IOR across a range of conditions.

### Implications on the “Two-Form versus Two-Component” Debate

It is generally assumed that the P1 component arises from extrastriate cortex (Luck et al., 1994) and reflects the attentional modulation of early sensory processing. In previous ERP studies of IOR, P1 reductions have often been observed for peripheral targets appearing at peripherally cued locations (see Prime & Ward, 2006; Satel et al., 2013), although at other times these reductions are conspicuously absent despite evidence for IOR in behavior (e.g., Prime & Jolicoeur, 2009a). Similar reductions of neural activation that can be traced to the visual cortices have been obtained in IOR studies using other imaging techniques (e.g., Anderson & Rees, 2011; Muller & Kleinschmidt, 2007), suggesting that IOR effects observed in cueing paradigms without eye movements may be the result of habituation of the visual pathway stimulated by the cue (Berlucchi, 2006; Dukewich, 2009; Satel et al., 2011). Multiple components theory predicts that P1 modulations would only occur when both the cue and the target are peripheral onsets and occupy the same retinal location, as was the case in the peripheral cue condition of the present experiment. Thus, this theory would not predict any P1 reduction in the central cue condition because the peripheral targets appeared in a location that was not stimulated by the cue. Although the data in this sense are largely consistent with a two-component sensorimotor theory, we would be remiss to ignore our previous failure (Satel et al., 2013) to find a correlation between the target-elicited P1 reductions and the magnitude of the IOR effect in conditions requiring oculomotor responses to peripheral cues. Thus, precisely how or even whether the cue-modulated P1 reductions are responsible for greater IOR in the peripheral cue condition cannot be confidently inferred.

Indeed, an important observation from Taylor and Klein (2000) that gave rise to the two-forms theory of IOR was that, in any condition in which a saccadic response was required to either the cue or target, the magnitude of the IOR effect was never any greater when measured by peripheral as compared to central targets. Because no additional costs were observed in those conditions in which the sensory pathways were repeatedly stimulated, the implication was that the effect of IOR in conditions in which oculomotor responses to peripheral inputs were permitted were more likely to be related to output-based processes than to input-based processes (see also Hilchey et al., 2012; Hilchey et al., 2014). When applied to the two conditions tested in the present experiment, the two-forms theory predicts that the IOR effect should be comparable in the peripheral and central cue conditions. At first glance, the present pattern of behavioral results seems to contradict such a prediction and be more in line with theories claiming that IOR comprises independent sensorimotor processes (e.g., Abrams & Dobkin, 1994; Satel & Wang, 2012; Wang et al., 2012). We speculate, however, that the discrepancy between Taylor and Klein's (2000) behavioral findings and those presented here can be accounted for by a methodological difference. The current study intermixed peripheral and central saccade cues during experimental trials, and the IOR effect was measured by way of a manual response to peripheral signal. In contrast, Taylor and Klein (2000) mixed both saccade cues and manual targets during experimental trials. This subtle methodological difference is not trivial. In the present design, we deliberately ensured that the central arrow cues had no manual responses associated with them so as to create a condition in which IOR was unequivocally caused by oculomotor response activation. Previous work has demonstrated that IOR, or IOR-like effects, are spuriously generated when cues are associated with manual responses (Hilchey et al., 2013). The peripheral signals in the present design were associated with both oculomotor and key press responses. It thus remains unclear whether the statistically larger peripherally generated IOR effect is due to (a) a degradation of the input signal as seems plausible given the target-elicited P1 reductions, or (b) the combination of manual- and oculomotor-generated IOR effects to the saccade cue, which possibly accounts for why cue-induced enhancements of Nd were greatest at contralateral electrode sites in the peripheral cueing condition. Future work focused on systematically manipulating the presence of central arrow, and peripheral onset signals will be required to distinguish between these possibilities.

### Nd as a Potential Neural Marker for IOR

Clearly, considerable caution is required when interpreting whether increased behavioral IOR in the peripheral cue condition is the result of an additional input-based component (i.e., a degraded percept as a result of repeated stimulation along the input pathways). Despite this, we collected electrophysiological data from a condition (the central arrow cue condition) in which, true to the seminal framework for IOR, delayed responding to peripheral targets was achieved unambiguously by way of an oculomotor/orienting response to that location in space (and not repeated sensory stimulation). This particular form of IOR is much more likely to be an effect during natural search than the form generated and studied in covert spatial orienting paradigms. In the arrow cue condition, Nd was more positive when cued as compared to uncued, whereas no arrow cue-related modulations were observed for P1 and N1. Moreover, robust peripheral cue-related enhancements of Nd were observed at contralateral electrode sites. Thus, whereas cue-related modulation of the target-elicited P1 and N1 ERP components occurred only for peripheral cueing—despite behavioral IOR in both conditions—the cue-related increase in the later Nd was observed whether a saccadic eye movement was made to an arrow or peripheral cue, particularly at contralateral electrode sites. These results provide further evidence that P1 modulations are neither necessary, nor sufficient, for the behavioral observation of IOR and suggest that the contralateral Nd component is a more appropriate electrophysiological marker for the IOR effects we have observed here. Further work investigating the relationship between IOR and the Nd component (and beyond) in conditions where output-based forms of IOR occur is strongly encouraged so as to identify neural and electrophysiological markers of IOR in the form that it likely takes in the real world.

## Footnotes

1 An omnibus ANOVA on P1s with factors condition, cueing, and laterality revealed a main effect of condition, *F*(1,19) = 8.019, *p* = .011, , but no main effects of cueing, *F*(1,19) = 0.629, *p* = .438, , or laterality, *F*(1,19) = 0.206, *p* = .655, . The interaction of Cueing × Laterality was significant, *F*(1,19) = 5.840, *p* = .026, , but the interactions of Cueing × Condition, *F*(1,19) = 1.427, *p* = .247, , and of Condition × Laterality, *F*(1,19) = 0.542, *p* = .471, , were not significant. The three-way interaction Condition × Cueing × Laterality was also not significant, *F*(1,19) = 2.115, *p* = .162, .

2 An omnibus ANOVA on N1s with factors condition, cueing, and laterality revealed a main effect of condition, *F*(1,19) = 7.101, *p* = .015, , but no main effects of cueing, *F*(1,19) = 0.336, *p* = .569, , or laterality, *F*(1,19) = 0.001, *p* = .972, . The interaction of Condition × Laterality was marginally significant, *F*(1,19) = 3.553, *p* = .075, , but the interactions of Cueing × Condition, *F*(1,19) = 0.012, *p* = .912, , and Cueing × Laterality, *F*(1,19) = 0.982, *p* = .334, , were not significant. The three-way interaction of Condition × Cueing × Laterality was also not significant, *F*(1,19) = 1.408, *p* = .250, .
